# Delayed sciatic nerve branch injury following a gluteal stab wound presenting as intrinsic foot muscle denervation: A case report

**DOI:** 10.1016/j.radcr.2026.04.021

**Published:** 2026-05-05

**Authors:** Bahman Rasuli, Elaheh Zaremehrjerdi, Milad Haji Agha Bozorgi, Ali Forat Yazdi

**Affiliations:** a Department of Radiology, Arman International Hospital, Jame Jam Imaging Center, Tehran, District 3, Seoul St, Q9CW+GH3, Iran; b Tehran University of Medical Sciences, Faculty of Medicine, Tehran, Iran; c Department of Orthopedics, School of Medicine, Iran University of Medical Sciences, Tehran, Iran; d Department of Radiology, School of Medicine, Advanced Diagnostic and Interventional Radiology Research Center, Iran University of Medical Sciences, Tehran, Iran

**Keywords:** Sciatic nerve injury, Peripheral nerve injury, Muscle denervation, Intrinsic foot muscles, MRI, Penetrating trauma

## Abstract

Penetrating injuries to the gluteal region may result in occult peripheral nerve damage. Delayed presentation and nonspecific symptoms can lead to missed or late diagnosis, increasing the risk of irreversible muscle denervation and functional impairment. A 40-year-old previously healthy man presented with progressive left foot paresthesia and gait disturbance six months after sustaining a stab wound to the left buttock. Initial emergency management focused on hemorrhage control and wound closure, while early neurologic symptoms were overlooked. MRI of the left foot demonstrated diffuse edema and signal alteration within the intrinsic foot muscles, consistent with denervation injury. Retrospective clinical correlation revealed a penetrating gluteal injury, implicating a sciatic nerve branch injury as the underlying cause. This case emphasizes the importance of considering peripheral nerve injury in patients with penetrating trauma along the anatomic course of major nerve bundles. Early neurologic evaluation, appropriate imaging, and a multidisciplinary approach are essential to prevent delayed diagnosis and irreversible muscle atrophy.

## Introduction

Penetrating trauma to the pelvic and gluteal regions poses a significant risk of injury to major neurovascular structures due to their relatively superficial anatomical course [1,2]. The sciatic nerve, the largest peripheral nerve in the body, is particularly vulnerable in this region, where even low-energy penetrating injuries may result in partial or occult nerve damage [3].

Peripheral nerve injuries may present with delayed or progressive symptoms, especially when initial neurologic deficits are subtle or overlooked during acute trauma management [4]. MRI plays a crucial role in detecting indirect signs of nerve injury, such as denervation-related muscle edema and atrophy, particularly when the site of injury is remote from the patient’s presenting symptoms [5].

This report aims to highlight the role of MRI in detecting delayed denervation changes in intrinsic foot muscles following gluteal stab wounds and to emphasize the importance of correlating distal muscle findings with proximal nerve injury.

## Case presentation

A 40-year-old man was referred to our institution with a six-month history of progressive paresthesia in the left foot, which had recently worsened and resulted in significant difficulty with ambulation. He had no history of diabetes mellitus, peripheral vascular disease, smoking, alcohol use, or other systemic illness.

Six months earlier, the patient sustained a stab wound to the left buttock during a physical altercation. He was evaluated in the emergency department, where active bleeding was controlled and the wound was sutured. At that time, he reported tingling in the left foot; however, no detailed neurologic examination or imaging assessment was performed, and the symptom was attributed to local soft-tissue injury. Electrodiagnostic studies (EMG/NCS) were not performed at any point, which limits precise localization of the nerve injury and should be considered a limitation of this case.

No MRI or MR neurography was obtained immediately after the trauma. If imaging had been performed at that time, denervation-related changes would likely have been minimal or subtle, as acute muscle denervation may not produce significant edema immediately post-injury. This highlights the difficulty of detecting partial sciatic branch injuries in the acute phase.

Over the following 6 months, despite conservative management including physiotherapy which resulted in partial improvement of sensory symptoms the patient developed progressively worsening paresthesia and gait disturbance in the left foot. Laboratory investigations, including metabolic and inflammatory markers, were within normal limits. Physical examination by an orthopedic surgeon raised suspicion for neuropathic pain, which was considered atypical in the absence of predisposing conditions.

**Fig. 1:**
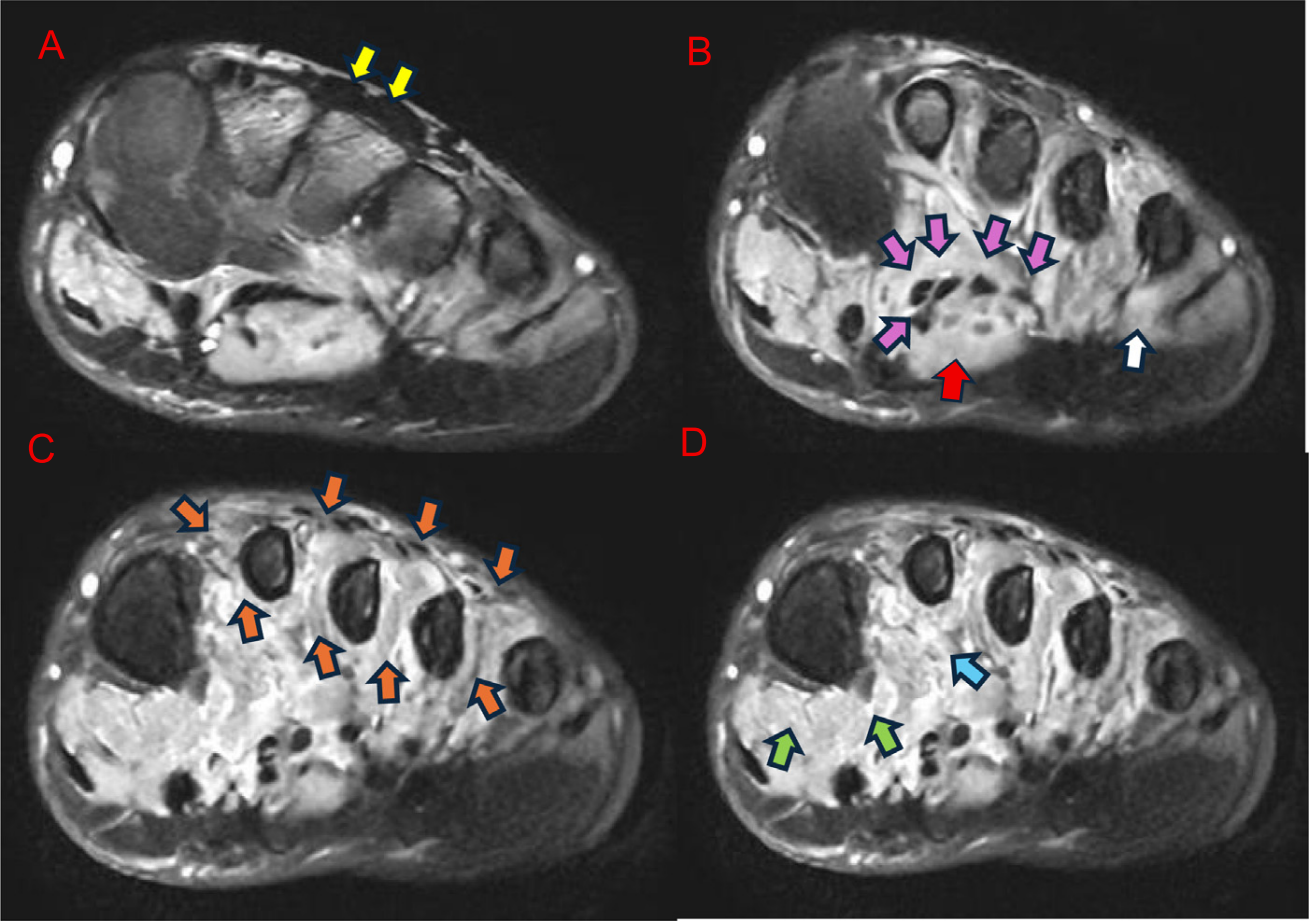

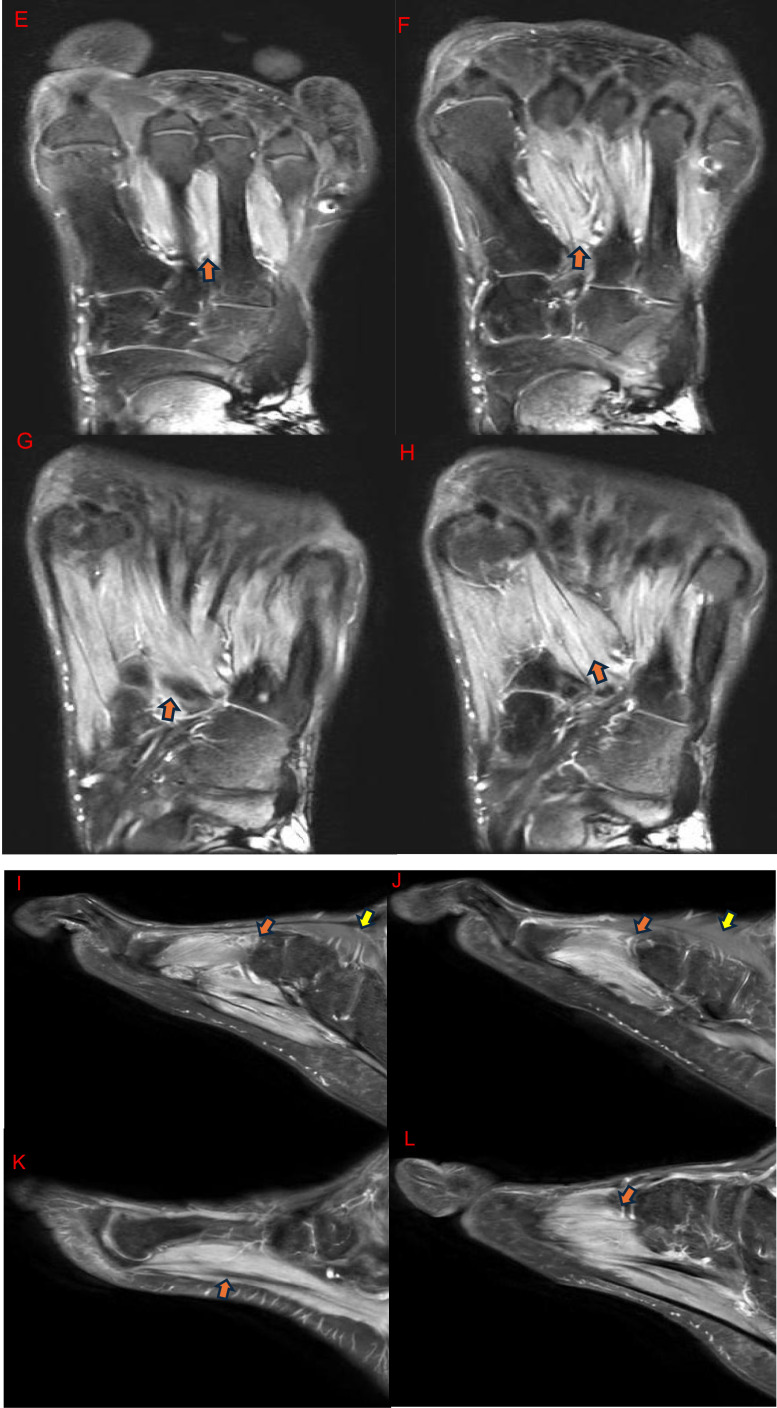
MRI of the left foot demonstrating denervation-related muscle edema. (A-D) Axial proton density (PD) fat-suppressed images show diffuse hyperintense signal involving multiple intrinsic foot muscles within the plantar compartments and intermetatarsal spaces including plantar and dorsal interossei (orange arrows), flexor hallucis brevis medial and lateral heads (green arrows), adductor hallucis (blue arrow), flexor digitorum brevis (red arrow), flexor digiti minimi (white arrow), lumbricals (purple arrows), while the dorsal intrinsic muscles (extensor digitorum brevis) appear unremarkable (yellow arrows). (E-H) Coronal proton density (PD) fat-suppressed images demonstrate diffuse edema across adjacent intrinsic muscle groups including planar and dorsal interossei (orange arrows) without focal mass, abscess, or direct muscular injury. (I-L) Sagittal proton density (PD) fat-suppressed images show diffuse plantar intrinsic muscle edema including planar and dorsal interossei (orange arrows), with preservation of the dorsal musculature including extensor digitorum brevis (yellow arrows), consistent with denervation secondary to proximal nerve injury.

MRI of the left foot was performed on a 1.5T system using axial, coronal, and sagittal proton density fat-suppressed sequences. No contrast was administered. Imaging demonstrated diffuse hyperintense signal involving the plantar and dorsal interossei, flexor hallucis brevis (medial and lateral heads), adductor hallucis, flexor digitorum brevis, flexor digiti minimi, and lumbricals, without evidence of focal mass, abscess, fracture, or direct muscular injury (Fig. 1). This pattern was consistent with acute-to-subacute denervation-related muscle edema.

No primary pathology of the foot itself was identified. Based on the characteristic MRI appearance, a proximal nerve injury was suspected. Retrospective review of the patient’s history revealed the prior penetrating injury to the left buttock, correlating with the anatomic course of the sciatic nerve and supporting the diagnosis of delayed sciatic nerve branch injury.

## Discussion

Peripheral nerve injuries following penetrating trauma are frequently underrecognized, particularly when immediate life-threatening conditions dominate the initial clinical assessment [2,4]. In the gluteal region, the sciatic nerve and its branches are especially vulnerable due to their superficial location and proximity to common sites of penetrating injury [1,3]. Minor penetrating trauma may result in partial nerve laceration or contusion, leading to delayed and progressive neurologic symptoms.

Given the predominant involvement of the plantar intrinsic foot muscles, the imaging findings are most consistent with injury to the tibial division of the sciatic nerve; however, the exact level and branch of nerve injury could not be definitively established without dedicated neurophysiologic or neurographic studies.

Several similar cases have been reported in the literature describing delayed sciatic or peripheral nerve injuries following penetrating or low-velocity trauma to the gluteal region. Kim et al. reported sciatic nerve injuries caused by penetrating trauma in which initial neurologic symptoms were mild and progressed over time due to incomplete nerve disruption [5]. Stewart et al. similarly described delayed-onset sciatic neuropathy following pelvic and gluteal trauma, emphasizing that early symptoms may be nonspecific and easily overlooked [3].

MRI features are highly valuable in distinguishing denervation-related edema from other causes of intrinsic foot muscle hyperintensity. In acute and subacute denervation, edema typically respects the anatomical compartment, involves expected muscle groups along the nerve distribution, and is diffuse without focal mass, abscess, or inflammatory changes. In our case, this pattern was consistent with acute-to-subacute denervation related muscle edema, as described in prior imaging studies [[6], [7], [8]]. Preservation of unaffected muscles, absence of contrast enhancement, and correlation with clinical history can further differentiate denervation from inflammatory myopathies, infection, or trauma-related muscle injury [[6], [7], [8]]. Lee et al. demonstrated that acute and subacute denervation manifests as muscle edema and T2 hyperintensity, whereas chronic denervation leads to fatty infiltration and muscle atrophy [6]. Fleckenstein et al. correlated MRI muscle signal changes with the timing and severity of nerve injury, supporting MRI as a reliable modality for detecting denervation before irreversible changes occur [8].

The differential diagnosis of intrinsic foot muscle edema includes peripheral neuropathy (e.g., diabetic or hereditary), inflammatory myopathies, and compartment-related pathology. In this patient, metabolic, inflammatory, and systemic causes were excluded based on laboratory investigations and clinical context, supporting denervation as the primary etiology.

Delayed recognition of peripheral nerve injuries has been associated with poorer outcomes, including persistent neuropathic pain and irreversible muscle atrophy [4,5]. Early multidisciplinary evaluation involving radiologists, orthopedic surgeons, neurologists, and rehabilitation specialists is essential to ensure timely imaging, appropriate electrodiagnostic testing, and optimal management, thereby minimizing long-term functional impairment.

## Conclusion

Penetrating injuries to the gluteal region should prompt careful evaluation for associated peripheral nerve damage, even in the absence of immediate neurologic deficits. MRI is invaluable for detecting denervation-related muscle changes when clinical presentation is delayed or nonspecific. Early recognition, multidisciplinary collaboration, and appropriate management are essential to prevent irreversible neuromuscular sequelae.

## Patient consent

The patient provided written informed consent for the publication of this case report, including all clinical data and accompanying images.
